# Robust calculation of slopes in detrended fluctuation analysis and its application to envelopes of human alpha rhythms

**DOI:** 10.1038/s41598-019-42732-7

**Published:** 2019-04-19

**Authors:** Guido Nolte, Mohammed Aburidi, Andreas K. Engel

**Affiliations:** 1 Department of Neurophysiology and Pathophysiology, University Medical Center Hamburg-Eppendorf, Hamburg, Germany; 2 Palestinian Neuroscience Initiative, Al-Quds University, Abu Dis, Jerusalem, Palestine

**Keywords:** Dynamical systems, Scientific data

## Abstract

Detrended fluctuation analysis (DFA) is a popular method to analyze long-range temporal correlations in time series of many different research areas but in particular also for electrophysiological recordings. Using the classical DFA method, the cumulative sum of data are divided into segments, and the variance of these sums is studied as a function of segment length after linearly detrending them in each segment. The starting point of the proposed new method is the observation that the classical method is inherently non-stationary without justification by a corresponding non-stationarity of the data. This leads to unstable estimates of fluctuations to the extent that it is impossible to estimate slopes of the fluctuations other than by fitting a line over a wide range of temporal scales. We here use a modification of the classical method by formulating the detrending as a strictly stationary operation. With this modification the detrended fluctuations can be expressed as a weighted average across the power spectrum of a signal. Most importantly, we can also express the slopes, calculated as analytic derivatives of the fluctuations with respect to the scales, as statistically robust weighted averages across the power spectra. The method is applied to amplitudes of brain oscillations measured with magnetoencephalography in resting state condition. We found for envelopes of the the alpha rhythm that fluctuations as a function of time scales in a double-logarithmic plot differ substantially from a linear relation for time scales below 10 seconds. In particular we will show that model selections fail to determine accurate scaling laws, and that standard parameter settings are likely to yield results depending on signal to noise ratios than on true long range temporal correlations.

## Introduction

Long-range temporal correlations are widely studied phenomena of brain dynamics measured from electrophysiological recordings like electroencephalography (EEG), magnetoencephalography (MEG) or local field potentials. A possible explanation of such long-range temporal correlation is the characterization of the complex nonlinear dynamical system as operating near criticality, and a prominent method to estimate long-range temporal correlation is the detrended fluctuation analysis (DFA)^1^. In DFA, data are divided into segments of length *L* and are linearly detrended. The square root of the variance (called fluctuation) of the detrended data is studied as a function of *L*. It can be shown that a linear relationship between the logarithm of the fluctuation and the logarithm of *L* is indicative of a power law behavior of the spectrum^2^. If such a linear relationship exists, the slope of the corresponding line is also referred to as Hurst exponent. In empirical data, the linear relationship cannot be detected over the full range of possible temporal scales *L*. The detection of a reasonable range to estimate a Hurst exponent is highly non-trivial, and a model selection scheme was suggested to arrive at valid conclusions^2^. A problem in particular for EEG and MEG data is the fact that the measured signals are superpositions of different brain sources with in general different dynamics. The effect of this mixing was studied by Blythe *et al*.^3^.

When applied to EEG or MEG data, DFA is mainly used to study amplitudes of brain oscillations. By now, a ubiquitous amount of literature exists not only to observe the phenomenon by itself^4,5^, but the estimated Hurst exponents are also used to distinguish patients from healthy controls, e.g. to study depression^6–8^, reading difficulties in children^9^, spastic diplegia^10^, and Alzheimer’s disease^11^. Differences in Hurst exponents were also found in different conditions, e.g. to track comprehension of speech^12^, performance of sensori-motor tasks^13^ and timing errors^14^, in perception of ambiguous visual stimuli^15^, and with medication^16^.

As will be outlined more formally below, while the classical DFA method is very simple, this approach is, to our opinion, conceptually not convincing. Data points at the edge of a segment are detrended differently from those in the middle: for data points on the edge neighboring data points, which happen to be outside the segment, are ignored for the detrending which is not the case for a point in the middle of the segment. Thus, time points are treated differently depending on the relative position of an arbitrarily set segment. We call this a methodological non-stationarity which is not backed by non-stationarity of the data. The effect itself was also shown by Kiyono and Tsujimoto^17^, but there it was addressed to nonlinearity, rather than non-stationarity, of the classical DFA approach.

In this paper we use a simple remedy, which is equivalent to detrending moving average (DMA)^18,19^, but we will here view this as a stationary version of DFA. Most importantly, we will argue that DFA, if expressed in a stationary formulation, is always equivalent to DMA and differences are only due to how a local average is constructed. Furthermore, fluctuations can be calculated in the Fourier domain. The resulting expressions can be evaluated numerically with minimal computational cost and are statistically much more robust than the classical approach in particular when calculating changes of fluctuations for small changes of scales *L*. Fourier domain DFA methods have been suggested before^19–22^ in general using different approaches, and, with one exception, without calculation of the slopes. We will make a conceptional comparison in a separate section.

The paper is organized as follows. In section 2.1 the classical DFA, also referred as non-stationary DFA in this paper, is recalled including an illustration of the inherent non-stationarity. In section 2.2 we present the theory in the Fourier domain after a minor modification to make DFA stationary was introduced. This theory can be considered as a special case of a whole class out of which one proposed alternative, using a Gaussian kernel for detrending, is analyzed theoretically in section 2.3. A relationship to other approaches of DFA in the Fourier domain is presented in section 2.4.

Results for simulated and empirical data are presented in section 3. After the describing the MEG data and the preprocessing steps in section 2.5, we illustrate differences and similarities for the estimated fluctuations using the classical and the proposed new methods. The final results of the estimates of slopes of the fluctuations as a function of scale for various frequency bands extracted from MEG data is presented in section 3.2. Here, we will analyze envelopes of alpha rhythm in more detail. Particular questions will be whether model selection can reliably detect scaling laws and to what extent results depend on signal to noise ratios. We finally make concluding remarks in section 4.

With regard to the theoretical concepts and also several observations on the drawbacks of classical DFA, this paper overlaps with existing work on DFA. Rather than strictly separating novel and known results we will present the theory as a whole in a hopefully logical style and refer to existing literature for each point to the best of our knowledge.

## Methods

### Classical DFA

In this section we first recall the standard procedure for DFA. Let {*x*(*t*)} for *t* = 1, ..., *T* be a time series, then one first calculates the cumulative sum

1 $$y(t)=\sum _{t^{\prime} =1}^{t}(x(t^{\prime} )-c)$$

where the constant *c* is the expected value of {*x*(*t*)} estimated by its mean. This constant is irrelevant for the classical DFA as it corresponds to a linear term in the cumulative sum which will be removed in the linear detrend outlined next.

The new time series *y* is then divided into segments of length *L*, and the cumulative sum for the *k*th segment reads

2 $${y}_{k}(t)=y((k-1)L+t)$$

for *t* = 1, ..., *L*. For each segment, a linear detrend is applied

3 $${z}_{k}(t)={y}_{k}(t)-{a}_{k}-{b}_{k}t$$

with constants *a*_*k*_ and *b*_*k*_ found from a linear fit. Quite generally, in this paper *x*(*t*) refers to the original data, *y*(*t*) to its cumulative sum or cumulative integral, and *z*(*t*) to the detrended cumulative sum or integral. To denote Fourier transforms of these, as will be done below, we will place ^^^ on these variables.

For *K* segments of length *L* the fluctuation *F*(*L*) of *z* is defined by

4 $${F}^{2}(L)=\frac{1}{KL}\sum _{kt}{z}_{k}^{2}(t)$$

Ideally, *F*(*L*) is linear in a log-log plot, i.e. *F*(*L*) ~ *L*^α^, and the slope *α*, which is also referred to as Hurst exponent, is calculated.

**Figure 1:**
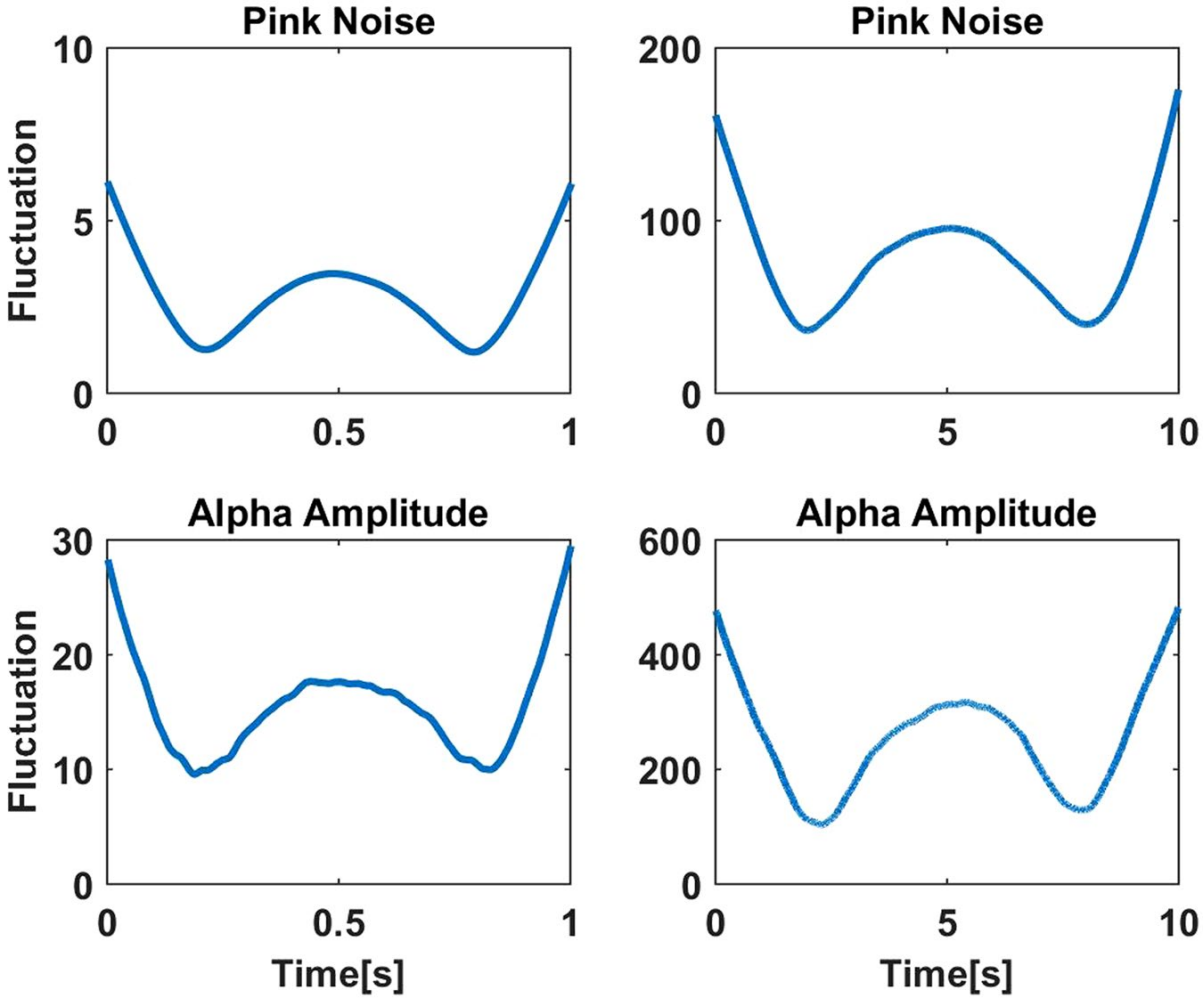
Fluctuations as a function of time relative to position within a segment for segments of length 1 second (left panels) and 10 seconds (right panels). Upper row: simulated pink noise. Lower row: empirical data example of the amplitude of alpha rhythm.

The above method treats time points differently depending on the position within arbitrarily set segments. E.g., for time points in the vicinity of the boundary nearby time points do not enter the detrending if they fall outside the segment. To show this, we calculated the fluctuation *G*(*L*, *t*) as a function of time within the segment as

5 $${G}^{2}(L,t)=\frac{1}{K}\sum _{k}{z}_{k}^{2}(t)$$

for simulated and empirical data. The data are explained in more detail in section 2.5 and are here used only for illustrative purposes. Results are shown in Fig. 1 where we observe that the fluctuation systematically depends on the time relative to the onset of the segments, with (local) maxima in the center of the segment and at the borders. Very similar results were also presented before but were addressed to presumably nonlinear properties of the DFA method^17^. To our opinion, the origin of this is rather non-stationarity than nonlinearity, which will be discussed in more detail, below.

This behavior can be qualitatively explained by time functions which are locally dominated by second order polynomials. If a linear detrend is removed in a specific segment of length 2*T*, the detrended signal has the form

6 $$f(t)=a({(t-T)}^{2}-{T}^{2}/3)$$

for 0 ≤ *t* ≤ 2*T* and with some constant *a*. What we observe is roughly the absolute value of that function.

Even though final results, namely the fluctuations, are barely affected by this on a wide scale (as we will show below empirically), this methodological non-stationarity is artificial and not based on a specific non-stationarity of the data.

### DFA in the Fourier domain

To avoid the non-stationarity described above we will here treat all time points equally and use a separate segment for each time point such that that time point is in the center of that segment. For each segment, detrending will then only be applied to the center of that segment.

With all time points being in the center of the segments (which requires an odd number of time points for each segment) a simplification occurs in the detrending: it is sufficient to remove a constant because the linear part orthogonal to a constant vanishes at the center. Thus we can write the detrended data as

7 $$z(t)=y(t)-\frac{1}{L}\sum _{\tau =-M}^{M}y(t+\tau )$$

with *L* = 2*M* + 1 and *y*(*t*) calculated from the data with Eq. 1.

This approach is identical to the detrending moving average (DMA)^17,18^ but viewed here as a consequence using DFA for the centers only, rather than a different method. Note that in the above equation values for *y*(*t*) have to be defined for *t* < 1 and *t* > *T*. For the latter we use periodic boundary conditions, and the fluctuations can now be expressed without splitting into segments simply as

8 $${F}^{2}(L)=\frac{1}{T}\sum _{t}{z}^{2}(t)$$

While in principle we could also simply not use time points near the boundaries of the data and avoid the need for boundary conditions, below we will reformulate DFA in the Fourier domain and then periodic boundary conditions are implicit. Such additional assumptions are potentially a caveat. However, if the mean is subtracted from the cumulative sum as defined in Eq. 1 we found that these boundary conditions do not cause a problem as will be illustrated in section 3.1.

Identical to formulations presented for DMA^19^, the methods proposed here are based on two basic relations. First, according to Parseval’s theorem we can express the fluctuation in the frequency domain as

9 $${F}^{2}(L)=\frac{1}{{T}^{2}}\sum _{f}{\hat{z}}^{2}(f)$$

where $\hat{z}(f)$ denotes the Fourier transform of *z*(*t*). An additional factor, here 1/*T*, can vary depending on the normalization of the Fourier transform. This factor does not affect the slope of log(*F*(*L*)).

Second, the construction of *z*(*t*) in Eq. 7 has the form of a convolution and can more formally be written as

10 $$z(t)=y(t)-({h}_{L}\,\ast \,y)(t)$$

where * denotes convolution, and *h*_*L*_(*t*) corresponds in this case to a boxcar window, i.e. *h*_*L*_(*t*) = 1/*L* for |*t*| ≤ *M* and zero otherwise. According to the convolution theorem

11 $$\hat{z}(f)=(1-{\hat{h}}_{L}(f))\hat{y}(f)$$

and the fluctuation reads

12 $${F}^{2}(L)=\frac{1}{{T}^{2}}\sum _{f}{(1-{\hat{h}}_{L}(f))}^{2}|\hat{y}(f){|}^{2}$$

The preceding equation is the general form to express fluctuations using arbitrary windows. For the case of a boxcar window ${\hat{h}}_{L}(f)$ can be calculated analytically:

13 $$\begin{array}{rcl}{\hat{h}}_{L}(f) & = & \sum _{t=1}^{T}{h}_{L}(t)\exp (\frac{2\pi ift}{T})\\  & = & \sum _{t=1-M-1}^{T-M-1}{h}_{L}(t)\exp (\frac{2\pi ift}{T})\\  & = & \frac{1}{L}\sum _{t=-M}^{M}\exp (\frac{2\pi ift}{T})\\  & = & \frac{1}{L}\sum _{t=-M}^{M}{(\exp (\frac{2\pi if}{T}))}^{t}\\  & = & \frac{1}{L}\frac{\sin (\pi fL/T)}{\sin (\pi f/T)}\end{array}$$

where the last step was done with the standard geometric series. Note that in the second step we have shifted the entire summation index by −*M* − 1 which does not affect the result because all functions are periodic.

In Eq. 12 we expressed the fluctuation in terms of the Fourier coefficients of the cumulative sum of the original data. We now construct an expression using directly the Fourier coefficients of the data. Writing the original data as $x(t)={\sum }_{f}\hat{x}(f)\exp (i2\pi ft/T)$ the cumulative sum reads

14 $$y(t)=\sum _{f}\hat{x}(f)\{\sum _{\tau =1}^{t}\exp (2\pi if\tau /T)\}$$

We recall that $\hat{x}(f)=0$ because the mean was subtracted from the data (see Eq. 1) and the term in curly brackets as for *f* ≠ 0:

15 $$\sum _{\tau =1}^{t}\exp (2\pi if\tau /T)=\frac{\exp \mathrm{(2}\pi ift/T)}{2\,\sin (\pi f/T)}a(f)-b(f)$$

with *a*(*f*) = exp(*iπf*/*T*)/*i* and *b*(*f*) = exp(*iπf*/*T*)/(2*i*sin(*πf*/*T*). Note, that this is a formulation in time domain and *b*(*f*) does not depend on time *t*. Hence, in the Fourier transformation of *y*(*t*) this term contributes to the *f* = 0 term. Thus, the cumulative sum of an oscillation is a scaled oscillation of the same frequency and a constant *b*(*f*). This constant part is irrelevant for the calculation of the detrended fluctuations because the weights vanish at *f* = 0. Also, the absolute value of *a*(*f*) is 1 and does not contribute to the power, and we can write the fluctuation as

16 $${F}^{2}(L)=\frac{1}{T}\sum _{f}{(1-\frac{1}{L}\frac{\sin (\pi fL/T)}{\sin (\pi f/T)})}^{2}\frac{|\hat{x}(f{)|}^{2}}{4{\sin }^{2}(\pi f/T)}$$

We are interested in the slope of log(*F*(*L*)) as a function of log(*L*). Rather than fitting a linear function of a finite range of scales *L*, we will here directly calculate the slope as a derivative with respect to log(*L*). This requires a remark on a technical detail. In the time domain, the stationary approach to calculate the fluctuations was only well defined for odd values of *L*. In the frequency domain we treat *L* as a free parameter which can assume any real value. We thus interpolate *F*(*L*) to arbitrary values for *L*. For such interpolations, one has to be careful how the Fourier transformation is defined. Without such interpolation, the full frequency range can be defined equivalently using negative frequencies or frequencies above the Nyquist frequency. E.g. for frequency *f* = 1, the oscillation 2cos(2*πt*/*T*) can be expressed both as exp(*i*2*πt*/*T*) + exp(−*i*2*πt*/*T*) and as exp(*i*2*πt*/*T*) + exp(*i*2*π*(*T* − 1)*t*/*T*). However, for the second expression this is only true for integer *t* and an interpolation to arbitrary *t* can only be done with the first expression. Using a convention for Fourier expansions with negative frequency leads to equal contributions to the fluctuations from negative and positive frequencies and one can just sum the positive frequencies with an additional factor 2 in the end. It is implicitly understood that in all expressions below sums over frequencies run up the Nyquist frequency. E.g., using Matlab convention it would be a severe mistake to sum over all frequencies including those above the Nyquist frequency.

With that remark made, we can calculate the desired slope which is the final formula for the boxcar window. Using *dL*/*d*log*L* = *L* the calculation is straight forward:

17 $$\frac{d\,\mathrm{log}(F(L)}{d\,\mathrm{log}(L)}=\frac{1}{{F}^{2}(L)}\sum _{f}{g}_{B}(f)\frac{|\hat{x}(f{)|}^{2}}{4{\sin }^{2}(\pi f/T)}$$

with

18 $${g}_{B}(f)=L(1-\frac{\sin (\pi fL/T)}{L\,\sin (\pi f/T)})(\frac{\sin (\pi fL/T)}{{L}^{2}\,\sin (\pi f/T)}-\frac{\pi f\,\cos (\pi fL/T)}{L\,\sin (\pi f/T)})$$

### The Gaussian window

In the preceding section the detrending was formulated as a subtraction of a local average of the cumulative sum of the data. This could be expressed as a convolution using a discrete boxcar window. Since the boxcar window has sharp edges, its Fourier transform falls off rather slowly with increasing frequencies. In this section we propose an alternative, namely to replace the boxcar window by a Gaussian window which decreases exponentially in the frequency domain with increasing frequency. A problem with the Gaussian window is that analytic calculations cannot be done for discrete and finite time, and we will use a formulation valid for continuous time instead also assuming an infinite length of data. In the end we will approximate the final integrals by sums.

For the Gaussian formulation of the detrending, *y*(*t*) is a function of all real valued time points *t*. Subtracting local mean with weights chosen from a Gaussian window of width *σ* leads for the detrended signal *z*(*t*) to

19 $$z(t)=y(t)-\frac{1}{\sigma \sqrt{2\pi }}{\int }_{-\infty }^{\infty }\exp (-\frac{{\tau }^{2}}{2{\sigma }^{2}})y(t-\tau )d\tau $$

Using the convolution theorem and standard results for the Fourier transform of the Gaussian function, the Fourier transform of *z*(*t*) reads

20 $$\hat{z}(f)=(1-\exp (\,-\,2{\pi }^{2}{f}^{2}{\sigma }^{2}))\hat{y}(f)$$

and for the fluctuation

21 $${F}_{G}^{2}(\sigma )={\int }_{-\infty }^{\infty }{(1-\exp (-2{\pi }^{2}{f}^{2}{\sigma }^{2}))}^{2}|\hat{y}(f{)|}^{2}df$$

where the sub-index *G* refers to the Gaussian case.

Similar to the discrete case, we can also express $\hat{y}(f)$, the Fourier transform of the cumulative integral, by the Fourier transform of the original data. With

22 $${\int }_{0}^{t}\exp (i2\pi {f}_{0}\tau )d\tau =\frac{\exp (i2\pi {f}_{0}t)}{2\pi {f}_{0}}i+\frac{i}{2\pi {f}_{0}}$$

we see that the time integral of an oscillation of frequency *f*_0_ is an oscillation with the same frequency and different amplitude plus a constant. The constant and also the phase shift of the oscillation are irrelevant for the fluctuation which can then be expressed as

23 $${F}_{G}^{2}(\sigma )={\int }_{-\infty }^{\infty }{(1-\exp (-2{\pi }^{2}{f}^{2}{\sigma }^{2}))}^{2}\frac{|\hat{x}(f{)|}^{2}}{4{\pi }^{2}{f}^{2}}df$$

Note, that the difference between the discrete and the continuous case is only the factor 2*sin*(*πf*/*T*) in Eq. 15 which has now become 2*πf*. This corresponds to the limit *T* → ∞ and the replacement of *f*/*T* with integer frequencies *f* in the discrete version by the continuous frequencies *f*. Since in applications on actual data we will approximate the integral over the frequency by a discrete sum, anyway, also using the discrete version of the cumulation is a reasonable approach. However, to discuss general methods in the frequency domain, as we will do in the next section, the continuous formulation is more convenient.

Analogous to the boxcar window we can now also give an expression for the slope of *F*(*σ*) in a log-log plot:

24 $$\frac{d\,\mathrm{log}({F}_{G}(\sigma ))}{d\,\mathrm{log}(\sigma )}=\frac{1}{{F}_{G}^{2}(\sigma )}{\int }_{f}{g}_{G}(f)\frac{|\hat{x}(f{)|}^{2}}{{f}^{2}}$$

with

25 $${g}_{G}(f)={\sigma }^{2}{f}^{2}(1-\exp (\,-\,2{\pi }^{2}{f}^{2}{\sigma }^{2}))\exp (\,-\,2{\pi }^{2}{f}^{2}{\sigma }^{2})$$

For the Gaussian window we expressed the length scale by *σ*, the standard deviation of a corresponding Gaussian probability density. This scale is not directly comparable to *L*, the width of the boxcar. A uniform distribution of width *L* has a standard deviation of $L/\sqrt{12}$. All results in the next sections will be given as a function of *L*, and it is implicitly understood that a result for a given *L* using a Gaussian window is calculated with the setting $\sigma =L/\sqrt{12}$. Finally, when integrals are approximated by sums over integer frequencies *f*, the *f* in Eq. 23 and Eq. 25 is replaced by *f*/*T* for *T* time points of the whole data.

### Relation to other methods

The fluctuation using the Gaussian window and a continuous time formulation has the form (see Eq. 23)

26 $${F}_{G}^{2}(\sigma )=\int h(\sigma f)\frac{|\hat{x}(f{)|}^{2}}{{f}^{2}}df$$

Note, that *h* is a function of the product of *σ* and *f*. Let us analyze the case where $< |\hat{x}(f{)|}^{2} >$, with <> denoting expectation value, follows a power law, i.e.

27 $$ < |\hat{x}(f{)|}^{2}\, > \sim {f}^{-\gamma }$$

Then with the substitution *u* = *σf* we can calculate the expected value of the fluctuation as

28 $$ < {F}_{G}(\sigma )\, > \sim {(\int h(\sigma f)\frac{1}{{f}^{2+\gamma }}df)}^{\mathrm{1/2}}=c{\sigma }^{\mathrm{(1}+\gamma \mathrm{)/2}}$$

where $c=\int h(u)/{u}^{2+\gamma }du$ is an irrelevant constant. E.g. for white noise, *γ* = 0, the fluctuation is linear in a double logarithmic plot and the slope has the value 1/2. For pink noise, *γ* = 1 and the slope is 1.

The important observation here is that to recover the proper linear behavior for a spectral density following a power law, it is sufficient that *h* is a function of the product *σf* provided that the integrals exist. This is indeed also the case for the boxcar window, where *L* takes the role of *σ* if formulated for continuous time which can be derived explicitly with lengthy calculations.

Our goal is now to revert the preceding derivations and to understand formulations given in the frequency domain as corresponding methods in the time domain. Whenever *h* is a non-negative function, we can also write it as

29 $$h(f)={(1-\tilde{h}(f))}^{2}$$

with

30 $$\tilde{h}(f)=1-\sqrt{h(f)}$$

We can then use the convolution theorem in inverse direction and express the corresponding formulation in the time domain as a detrending subtracting a local weighted average from the cumulative sum. In other words, different methods of such a form are all stationary and correspond to different choices for the windows in the time domain, which are the inverse Fourier transforms of $\tilde{h}(f)$.

Several methods with the form of Eq. 26 with different choices of *h* have been suggested. We will discuss them shortly with minor deviation of language to avoid confusing terminology. Probably the simplest approach was proposed by Chianca *et al*.^21^ suggesting to exclude frequencies below a scale 1/*L*. This means that $\tilde{h}(f)$ is a boxcar, and the inverse Fourier transform, i.e. the window in the time domain, is a sinc function which is oscillating and converges to zero for large time differences rather slowly. Although one could in principle also derive an expression for the slope for a given scale, the final expression contains a delta-function and a numerical approximation is surely not very robust in a statistical sense because the result will be calculated from a single frequency.

Willson *et al*.^20^ make a low order Taylor expansion of the (classical) detrended cumulative sum with separate expansions for oscillations with frequencies smaller or larger that 1/*L*. In the language used here, this leads to a discontinuous $\tilde{h}(f)$. To our knowledge, this is the only work where also a formula for the slope is derived. However, the discontinuity is apparently a problem, and the authors suggest to ignore the contribution from it.

Probably the approach closest to the one presented here was proposed by Kiyono^22^. The author starts with the original non-stationary formulation and detrends the cumulative sum for various orders followed by an averages across phases. The rather complicated expressions also have the form given in Eq. 26. Hence, such formulations effectively ‘stationarize’ the method. For order 0, i.e. only a constant is removed in each segment of length *L* within the non-stationary formulation, the author finally arrives at (Eq. B2 of that paper ignoring global constants and with modifications to adjust to our terminology)

31 $${h}_{0}(f)=\frac{2{\pi }^{2}{f}^{2}{L}^{2}+\,\cos \,\mathrm{(2}\pi fL)-1}{{f}^{2}{L}^{2}}$$

and for order 1 the result is (Eq. B4 with modifications)

32 $${h}_{1}(f)=\frac{2{\pi }^{4}{f}^{4}{L}^{4}-4{\pi }^{2}{f}^{2}{L}^{2}-3+(3-2{\pi }^{2}{f}^{2}{L}^{2})\cos (2\pi fL)+6\pi fL\,\sin \,\mathrm{(2}\pi fL)}{{f}^{4}{L}^{4}}$$

**Figure 2:**
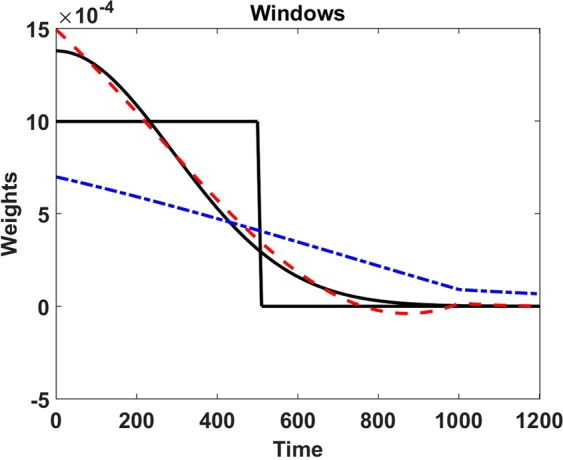
For a length scale of *L* = 1000, windows corresponding to order 0 and order 1 are calculated from Eq. 31 and Eq. 32 and are shown as blue dash-dotted and red dashed lines, respectively. For comparison we show the boxcar and the Gaussian window as black full lines with obvious distinction. Note that the windows are symmetric and we show only half of them.

The corresponding windows for *h*_0_ and *h*_1_ given in Eq. 31 and Eq. 32 cannot be calculated analytically. In Fig. 2 we show numerical results for segments of length *L* = 1000 including the boxcar and Gaussian window for comparison. We observe that for order 0, i.e. for *h*_0_, the window decreases almost linearly up to twice the segment length, and then decreases more slowly for larger delays. For order 1 the window is similar to the Gaussian window but becomes slightly negative, and it decreases more slowly than the Gaussian window.

The key observation here is that DFA is equivalent to DMA, and the only question is how the local averages are defined, i.e. the choice of window. While DFA is sometimes considered as a nonlinear method^18,19^, we here argue that a detrend is a linear operation which can be expressed as a linear filter if the same detrending operation is applied for all time points. The problem of classical DFA without overlapping windows is that data of neighboring time points are detrended differently, and that is a non-stationarity. The seemingly nonlinear properties of DFA can be avoided using maximally overlapping windows. If the segment length is *L*, then each point is detrended *L* times with *L* different detrending operations, but those are identical for all time points and the total fluctuations can be expressed as sums over *L* different linear filters. While such maximally overlapping windows indeed stabilize the results, the computational cost of calculations in the time domain would be extremely high^17^.

### Data and Preprocessing

We used measurements of MEG (CTF system with 274 sensors) in resting state with eyes closed for around 10 minutes in 40 subjects. The data were collected for a different project. Written informed consent was obtained by all participants prior to participation. The local Ethics Committee (Ethik-Kommission der Ärztekammer Hamburg) approved all methods, and all procedures were performed in accordance with the Declaration of Helsinki.

All subjects, except one, were measured twice in consecutive weeks, and we had a total 79 data sets. The data were measured with a sampling rate of 1200 Hz and were downsampled to 300 Hz. The primary goal here was the analysis of the amplitude of the alpha rhythm at around 10 Hz, and the following preprocessing steps were selected according to this.

1. For each data set we calculated the cross-spectrum for all pairs of channels in 1 Hz frequency resolution and averaged that across all frequencies within the alpha band between 8 and 13 Hz. We performed a PCA decomposition of the real part of that averaged cross-spectrum and reduced the dimension of the data by projecting them onto to the space, which was spanned by the 100 largest PCA components. The real part corresponds to the covariance matrix of the data filtered in that band and ensures that the eigenvectors are real valued such that the projected data are again real valued. Note, that the spatial projector is constructed from the alpha band only, but it is applied on white band data. The idea is to reduce the dimension of the data in such a way that the alpha rhythm is least distorted.
2. We then filtered these spatially projected data in a wide band between 5 and 45 Hz, and performed an ICA decomposition using the TDSEP algorithm^23^. Note, that for a separation of independent components which are not related to alpha rhythm also information outside the alpha band will be used. For each ICA component we calculated the power *P*(*f*) in 1 Hz frequency resolution.
3. In order to estimate the strength of the alpha peak, we estimated a noise level *N*(*f*) by a linear interpolation of powers at neighboring frequencies and a signal-to-noise ratio at frequency *f* with Δ*f* = 2 Hz as

33 $$N(f)=\frac{P(f+{\rm{\Delta }}f)+P(f-{\rm{\Delta }}f)}{2}$$

34 $$SNR(f)=\frac{P(f)-N(f)}{N(f)}$$

An ICA component was considered as containing substantial alpha rhythm if the maximum of *SNR*(*f*) within the alpha band was larger than 5. We considered this as a reasonable threshold to detect clear alpha rhythm, even though this threshold is somewhat arbitrary. A dependence on SNR will be presented below. This criterion left a total of 242 ICA components which were further analyzed. For each data set we compared the SNR of the best, i.e. highest SNR, with the SNR of the best channel. We found that using ICA components substantially improves the data with SNRs of the best ICA components being on the average three times larger than the average across all best channels.

**Figure 3:**
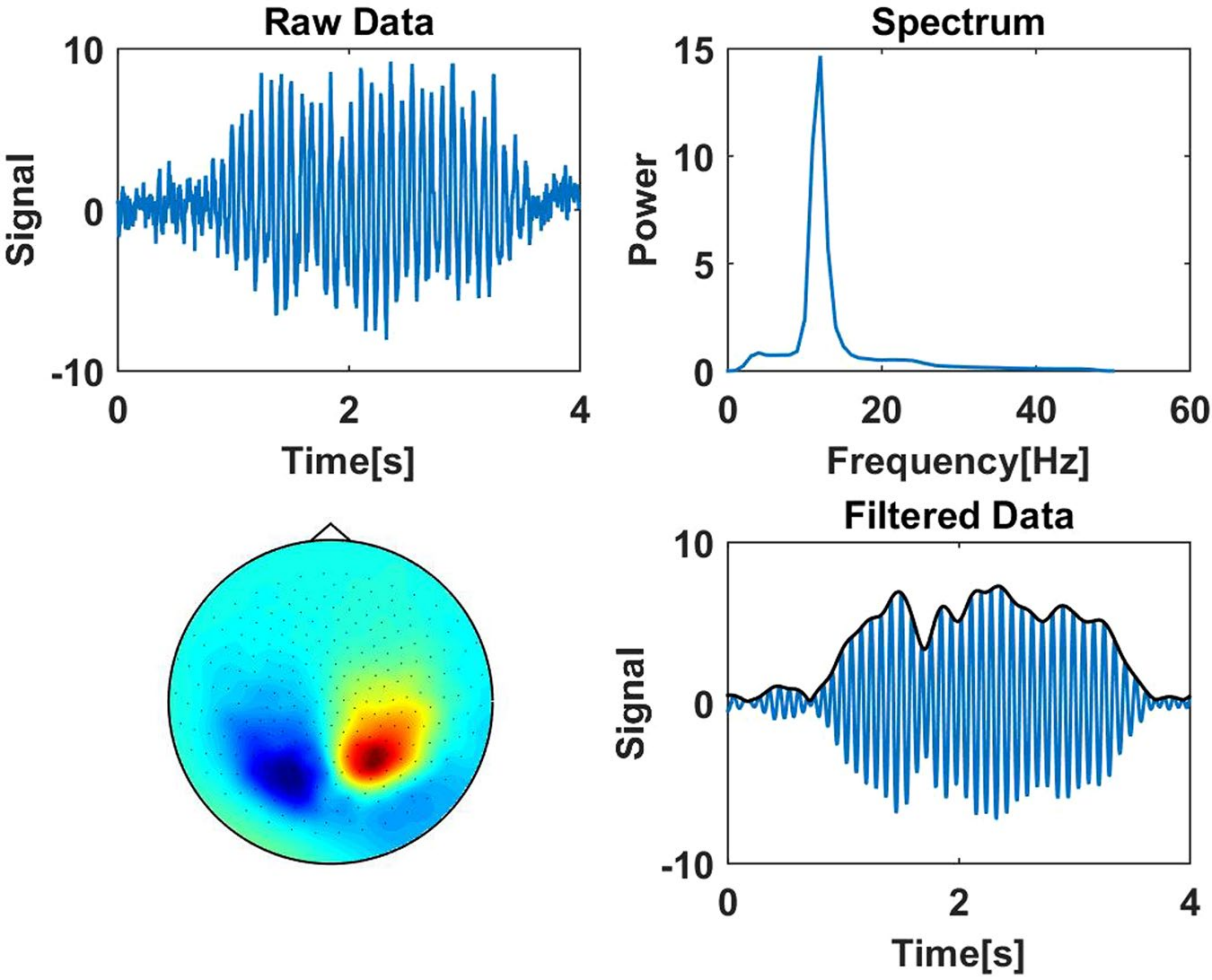
Upper left: Segment of raw data containing an alpha burst in an illustrative ICA component. Upper right: power spectrum of that ICA component with data filtered between 5 and 45 Hz. Lower left: Topography of the corresponding ICA component in sensor space. Lower right: Filtered data and envelope, shown as a black, curve. This envelope will be used in the following for DFA analysis. of all 242 ICA components selected as alpha rhythms.

A (nice) example of such an ICA component is shown in Fig. 3. There we show a 4 seconds snapshot of the raw data containing an alpha burst, the power spectrum of that component, and the spatial topography calculated from the ICA mixing matrix and the pseudo-inverse of the PCA projection. We observe a very clear peak in the alpha range and a dipolar topography over the occipital cortex. We also show the power spectra of all ICA components selected as alpha-rhythm.

## Results

### Non-stationary vs. stationary DFA

**Figure 4:**
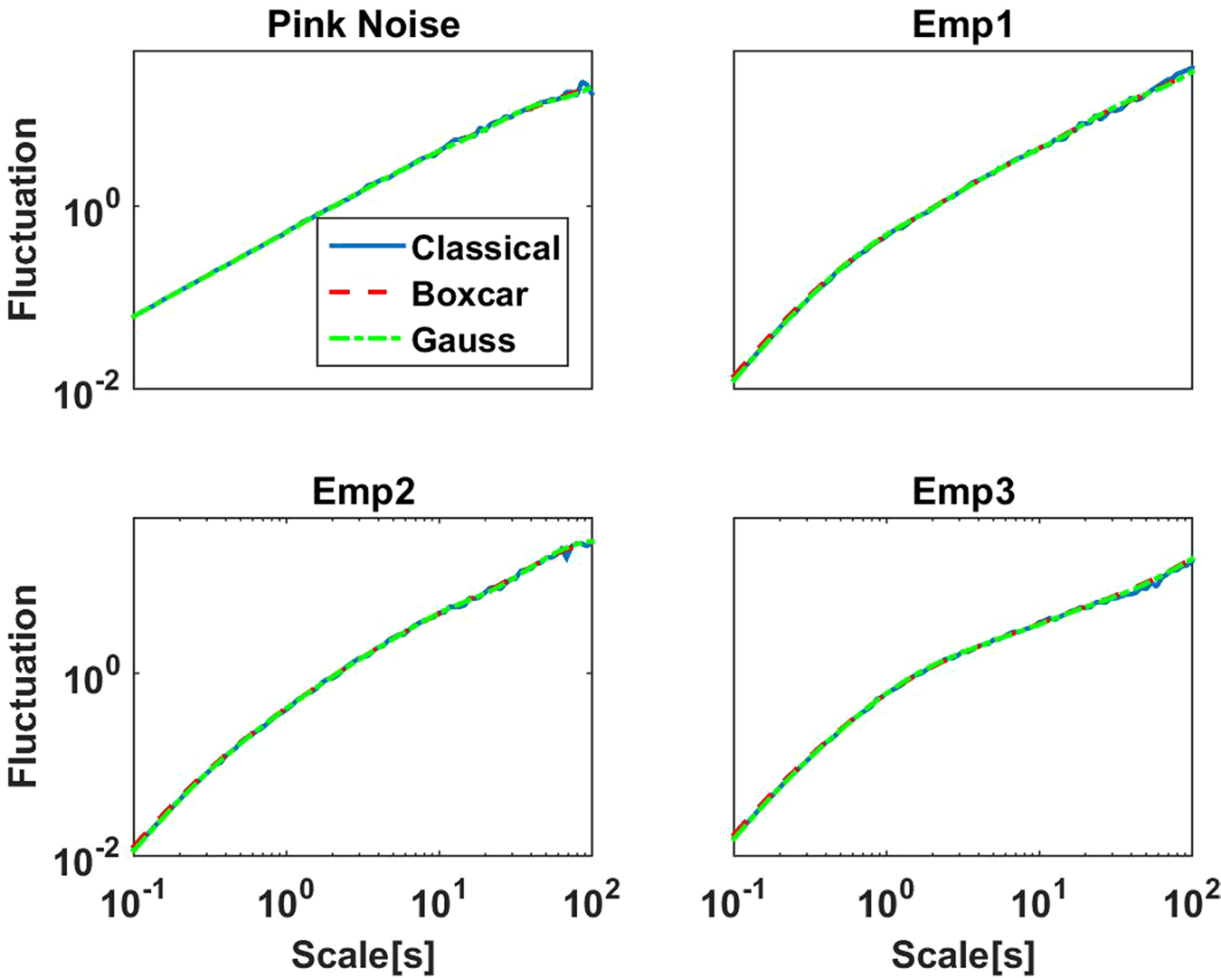
Illustrative examples showing fluctuations of detrended signals as a function of scale *L* for three different methods. The signals consisted of simulated pink noise (upper left) and of the amplitudes of alpha rhythm of three different ICA components.

We compare three different methods: a) the classical (non-stationary) DFA based approach, b) the Fourier method using a boxcar window, and c) the Fourier method using the Gaussian window. For simulated data with spectra following a power law, i.e. colored noise, log(*F*(*L*)) is always linear as a function of log(*L*) with the correct slope. For empirical data, the input for DFA are the amplitudes of the raw data filtered in the respective band (8 Hz to 13 Hz for alpha), with amplitudes calculated as the absolute value of the (complex) Hilbert transform of the filtered data. Fluctuations for empirical data in general deviate from linearity, and the question is whether the fluctuations differ for the three methods in such cases. First of all, fluctuations differ by a global factor, which, however, is irrelevant for the slopes of the logarithm of the fluctuations. To better compare the methods we normalized the fluctuations by the average of *F*(*L*) across values of *L* between 100 ms and 10 s. Results for simulated pink noise and three examples of the amplitude of alpha rhythm from MEG data, which are all very similar for alpha rhythm, are shown in Fig. 4, and we observe that fluctuations are almost identical across methods when viewed on such a wide range of scales *L*. We note, that these examples were not selected to show the desired result, but we found that this is generally the case.

**Figure 5:**
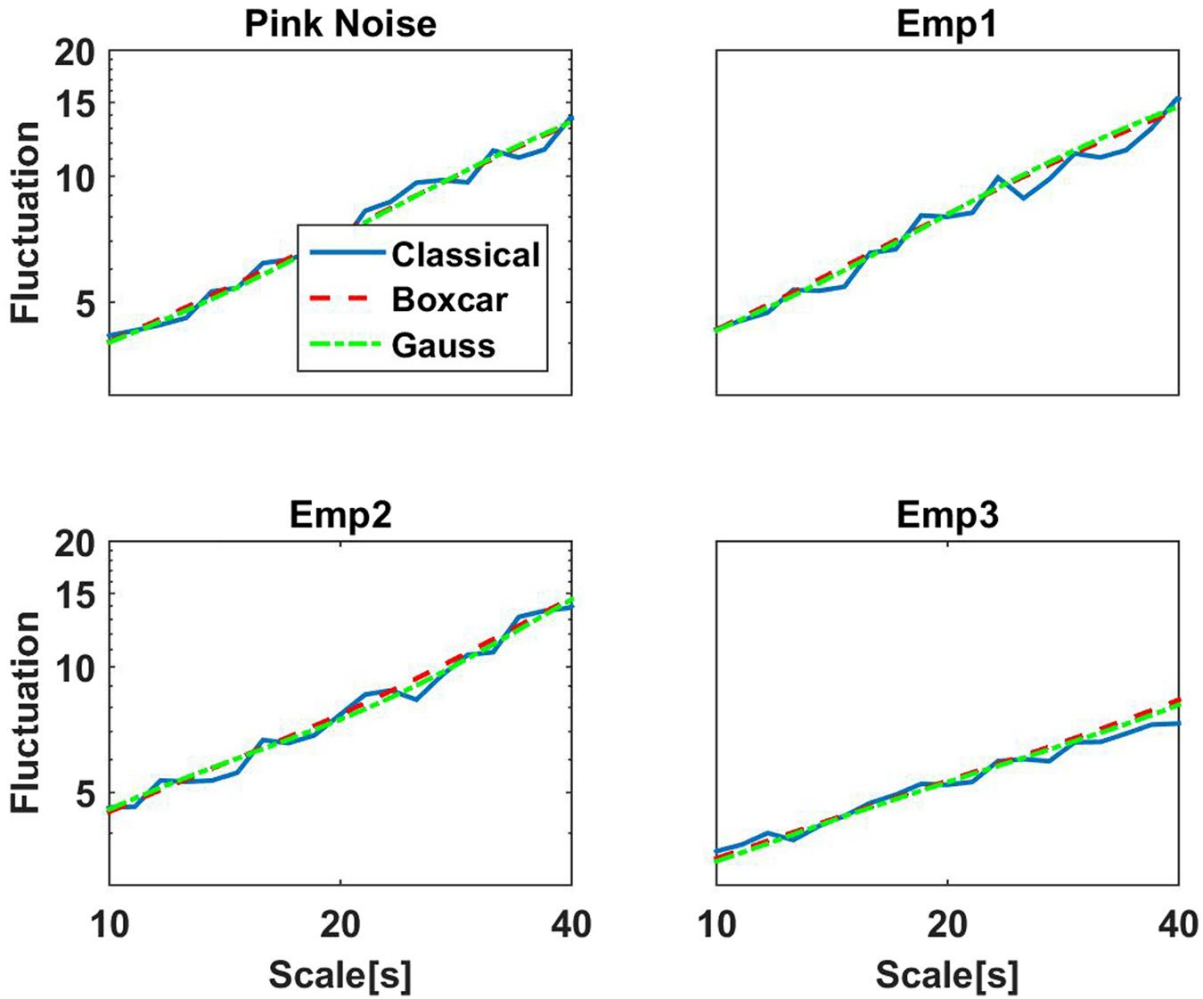
Same as Fig. 4 but now for scales between 10 and 40 seconds.

Deviations between the methods can be observed when looking at a smaller range of scales as shown in Fig. 5 showing fluctuations for scales between 10 and 50 seconds. The important observation here is that the classical method shows large variations of the fluctuations such that calculating the slope locally by a finite difference is apparently extremely unstable. The reason for that is the inherent non-stationary nature of the classical method which will be explained in more detail in the following.

**Figure 6:**
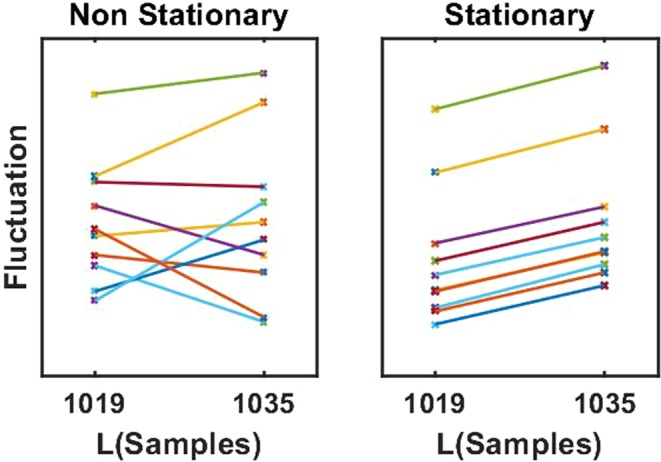
Fluctuations for two nearby scales calculated for 10 data sets of simulated pink noise for the classical approach (left) and for the proposed approach using the boxcar window (right). Results from the same data set are connected by lines.

To further illustrate the effects of local slope calculation and highlight the advantages of the proposed technique we simulated 10 data sets containing pink noise, each of 10 minutes duration with a sampling rate of 256 Hz, and calculated the fluctuations at two nearby values for *L*. This is shown in Fig. 6 both for the classical non-stationary approach and the proposed approach with the boxcar window. We observe that the variation of fluctuations is large relative to the systematic effect due to an increase of *L*. However, when connecting points with lines when they were calculated from the same data set, we see that the systematic effect can clearly be seen for the proposed approach but not in the classical one. The reason is that for the classical approach even a small change of *L* leads to very different detrending in particular for time points at the end of the data set: a point which was on the right side of a segment may turn out to be on the left side after a small change of *L* and the detrending is then based on very different data. In contrast, when detrending is always done in the center of each segment, changing *L* by a small value leads only to a small change of data used to calculate the detrended fluctuation for each time point. This situation is analogous to a paired versus unpaired statistical test. Even if the fluctuations vary across data sets, in the proposed approach the systematic trend as a function of *L* is clearly observable in each individual data set.

**Figure 7:**
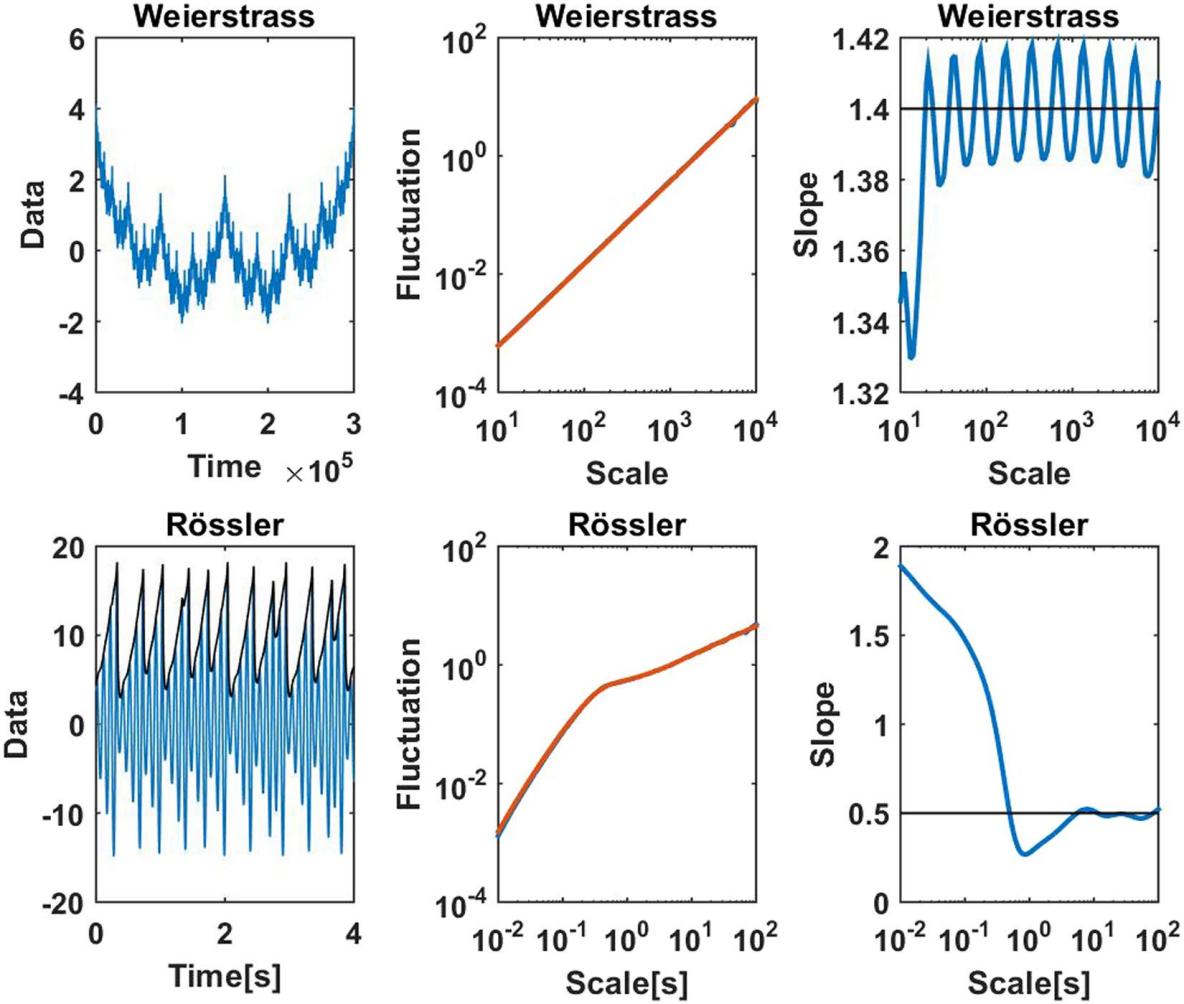
Upper panels: Data, fluctuations using the classical approach and the Fourier approach with boxcar windows, and the slope using boxcar window. The fluctuations were normalized to have equal mean. Fluctuations for both methods are shown but only a single line is visible because results are almost identical. Lower panels: 4 seconds of data, fluctuations and slope for the Rössler system. The input for DFA was the envelope of the data as shown by the black line in the lower left panel.

As final examples we apply the proposed on simulated nonlinear data. First of all, DFA can be used to estimate fractal dimensions in particular for the Weierstrass function^24^. We generated data corresponding to a fractal dimension of *D* = 1.6 for 300000 data points. The Hurst exponent *H*, which corresponds to the slopes in our analysis, is expected to relate to the dimension for this case as *H* = 3 − *D* = 1.4^24^. In the upper panels of Fig. 7 we show the full original data, the fluctuations calculated both with the classical method and the Fourier method using the boxcar window, and the slope using the boxcar window. The fluctuations appear largely linear and almost identical for the two methods. However, the slope is not constant but rather oscillates slightly around the value of 1.4. This reflects the fractal nature of this graph: its approximate self-similarity refers to discrete changes in scale and not to arbitrary changes. This effect is too small to be observable in the fluctuations. We note, that these oscillations for the slope are largely washed out when using the Gaussian window.

In a second example we applied our approach to the Rössler system, which is a chaotic dynamical system with fractal dimension^25^. Data were generated for the chaotic Rössler system using parameters as in^26^, and the time axis was re-interpreted such that the oscillations had a frequency of around 10 Hz. We generated 27 minutes of such data, calculated the envelope of the data, and calculated fluctuations and the slope, using the boxcar window, from that envelope. Results are shown in the lower panels of Fig. 7. Again we observe that the fluctuations are almost identical for the classical and proposed method. The slope, which is rapidly decaying to the white noise level of 0.5, shows that for this case long-range correlations do not exist.

### Analysis of Slopes for amplitudes of oscillations

#### The general picture

**Figure 8:**
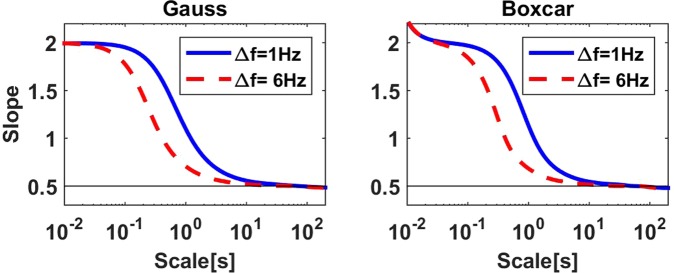
Slopes calculated from the amplitudes of filtered noise using a FIR filter with a length of 1 second. The band width was 6 Hz and 1 Hz, respectively.

For EEG and MEG data analysis, the typical application of DFA is the analysis of the amplitude of oscillatory activity. Before presenting results for empirical data, we first simulate such activity using filtered white noise. Alpha band in empirical data can be separated from other activity by filtering data in the alpha band chosen here between 8 and 13 Hz. In Fig. 8 we show slopes of log(*F*(*L*)) as a function of log(*L*) calculated with the proposed Fourier method using boxcar and Gaussian window for white noise sampled at 300 Hz, filtered in that relatively wide band, and with amplitudes calculated using the Hilbert transform. We used a FIR filter of 1 second duration. We observe that for very small scales in the order of 10 ms slopes start around 2 which we address to the large auto-correlation at small temporal delays due to the filtering. The slope declines mostly between 100 ms and 1 second and reaches the value of 0.5 after a few seconds.

Alpha rhythm typically has a more narrow spectral peak than the 6 Hz width chosen for the alpha band. To simulate the effect of the narrow peak itself we also narrowly filtered the white noise at 10 Hz again using a FIR filter of 1 second length such that the band width is about 1 Hz. In this case the decline of the slope as a function of scales *L* is shifted to larger scales such that the effect of that filter is substantial for scales below 10 seconds as can be seen in Fig. 8. We emphasize that this effect occurs even though the original simulated data were strictly uncorrelated for delays larger than 1 second corresponding to the length of the FIR filter.

**Figure 9:**
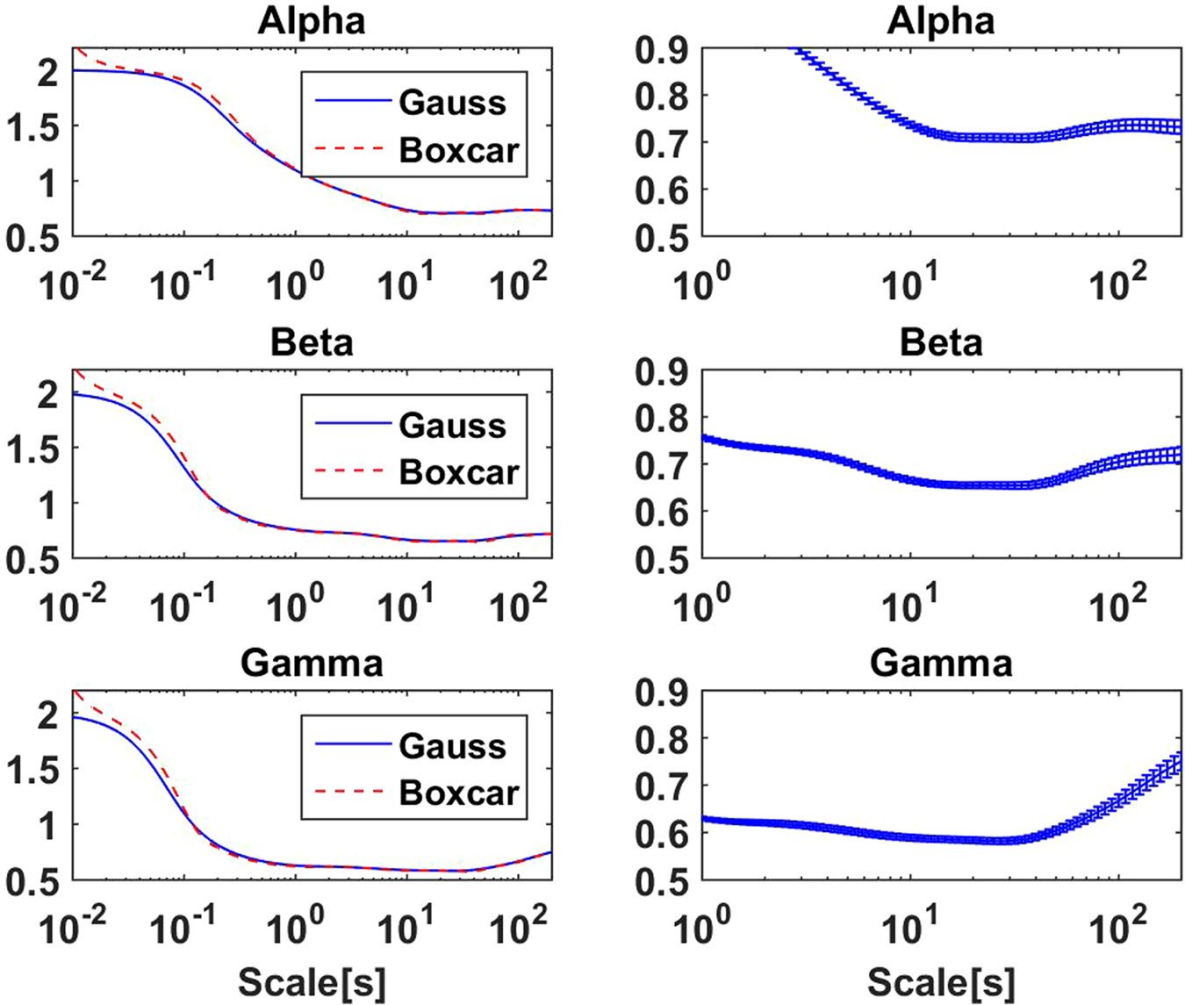
Slopes calculated with the proposed methods from empirical data from the amplitudes of band filtered ICA components. Left: slopes are shown for the full range of scales. Right: slopes are shown for the Gaussian window for scales larger than 1 second and including error bars calculated as standard error of mean. Results for the boxcar window are almost identical.

As explained in more detail in section 2.5, we analyzed the data of a total of 242 ICA components. Although the components were selected to show strong alpha rhythm, we used the same components also for beta range and for gamma range oscillations. For each frequency band, alpha between 8 and 13 Hz, beta between 15 and 30 Hz, and gamma between 30 and 48 Hz, we filtered the data in that band and calculated the amplitudes. Slopes of log(*F*(*L*)) were calculated from these amplitudes using the Fourier methods both with boxcar and Gaussian window for 130 different values of scale *L* between 10 ms and 200 seconds. Results are presented in Fig. 9. The left column of panels shows results over the complete range of scales. As for the simulated filtered noise we observe large slopes for small scales with a steady decrease with increasing *L*. With details depending on the band, the slopes are relatively constant for scales larger than 10 seconds. We also observe a slight increase of slopes above around 50 seconds most of all for the gamma range. The origin of this is not clear to us. Most importantly, the slopes for the alpha rhythm are substantially different from a constant for scales below 10 seconds. We address this to the narrow band spectral properties of typical alpha rhythms as discussed for the previous simulations.

In the right panels of Fig. 9 we show results only for scales above 1 second to have a closer look at the properties for scales relevant in practice. Results are only shown for the Gaussian window (boxcar is similar) but now including standard errors of the mean calculated as the standard deviation across all components divided by $\sqrt{242}$ corresponding to the number of components. This is based on the assumption that the ICA components can be considered is sufficiently independent, which will be discussed below in more detail. We refrained from estimating errors for individual components, because the objective of the study, the long range temporal correlation, is at least conceptually in contradiction to the requirement of independence, e.g. of individual segments, necessary for statistical analysis. We observe constant slopes clearly above 0.5 in particular between 10 and 50 seconds. Again, the most important result here is that slopes are in general not constant for scales below 10 seconds.

#### Model selection

**Figure 10:**
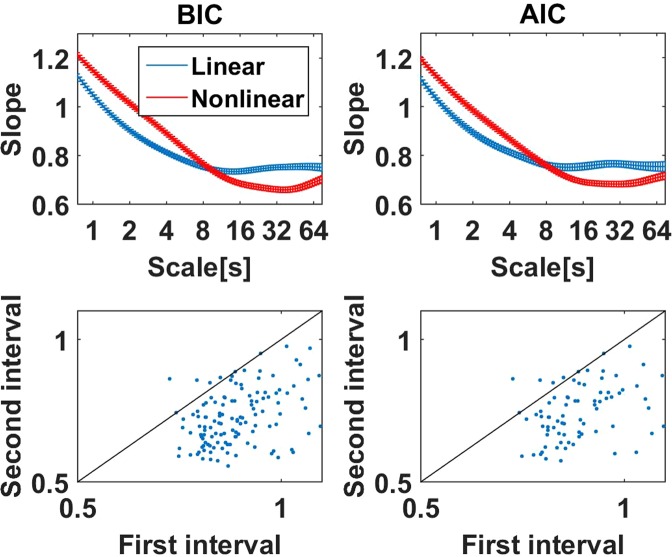
Top row: Slopes calculated for data, for which a linear/nonlinear behavior of the fluctuations (i.e. constant slope/non-constant slope) was found as the best model using BIC and AIC selection criteria. Bottom row: For all those data sets which were classified as linear, the entire region of time scales between 750 ms and 75 s was devided into two intervals, 750 ms to 7.5 s and 7.5 s to 75 s, respectively, and the slopes were calculated using a classical DFA in these intervals. We show the results of these slopes for both intervals for all these selected data sets.

Ton and Daffertshofer^2^ suggested to use a model selection based either on the Bayesian Information Criterion (BIC) or Akaike Information Criterion (AIC), and the software can be downloaded from Github (“FluctuationAnalysis”). In that software 11 different models for the fluctuations as a function of time scale are compared. We followed the proposed analysis^2^ and analyzed scales between 750 ms and 75 s using 100 equally distributed time scales on a logarithmic time axis. From a total 242 ICA components containing alpha rhythm with an SNR > 5 we found that for 128 components the linear model for the fluctuation (i.e. constant slope) was selected as the most likely model using the BIC criterion and 77 were selected using the AIC criterion. The average slopes (using a Gaussian window) of these selected ICA components (denoted as ‘linear’) are shown in Fig. 10 and can be compared to the corresponding results for all those components for a which some other model (denoted as ‘nonlinear’) was preferred. We observe that slopes for the linear cases are closer to a constant but the qualitative behavior is the same: the slopes show a systematic and substantial decrease up to scales of around 10 seconds. We further divided the entire region of scales into two intervals, [750 ms, 7.5 s] and [7.5 s, 75 s], and calculated the slopes from a classical DFA (i.e. without model selection) from all those components which were selected as linear in the full range. Results for all individual components are shown in the lower panels of Fig. 10. We observe that slopes in the first interval are systematically larger than in the second interval which would not be the case if the fluctuation functions were linear as was suggested by the model selection.

#### Dependence on signal to noise ratio

**Figure 11:**
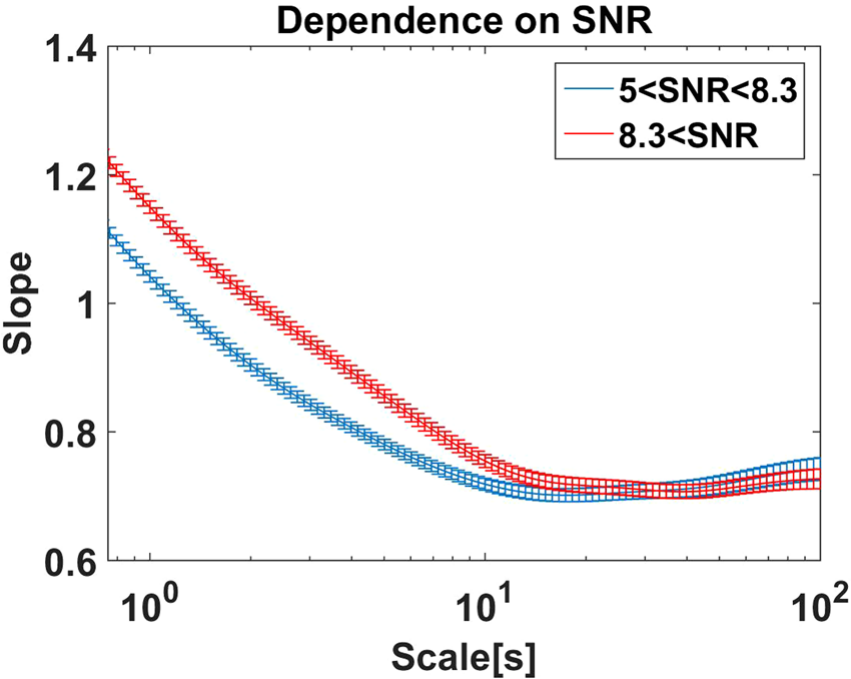
Slopes calculated for amplitudes of alpha oscillations for data with (relatively) low signal to noise ratio and high signal to noise ratio.

We finally analyzed whether slopes of fluctuation functions depend systematically on the signal to noise ratio. We divided the set of all 242 ICA components containing alpha rhythm with SNR > 5 into two groups of equal size with 5 < SNR < 8.3 and 8.3 < SNR, respectively. Results for the average slopes within each group including standard errors of the mean are shown in Fig. 11. We observe a systematic dependence on SNR for scales below approximately 10 seconds, while the saturation region for scales above 10 seconds is largely independent of the SNR for these relatively large SNRs. This shows that results for estimating Hurst exponents from scales starting at 1 or 2 seconds and indicating a difference between patients and healthy controls or different conditions could merely be a result of different signal to noise ratios. However, the good news is that such a misinterpretation can easily be avoided by choosing sufficiently large time scales.

#### A technical note on the estimation of standard errors of mean

If we have *N* independent observations of a quantity with standard deviation *σ*, then the average of those observations has a standard deviation $\sigma \,\sqrt{N}$, termed standard error of the mean (or just standard error). We used this formula for all figures where the standard error was included, which was based on the idea that all ICA components are reasonably independent of each other. However, the brain is an interacting system and ICA components are not statistically independent, and even though filtered raw data are almost uncorrelated across ICA components this effect is not negligible for the envelopes of alpha oscillations.

We were not able to find a correction scheme in the literature which would be applicable for a general case. We therefore derived a rather simple correction formula ourselves without claiming that it is new. The technical details are presented as supplementary information. The final result is that if we have observations *x*_*i*_ for *i* = 1, ..., *N* with mutual correlations *ρ*_*ij*_, then the standard error of the mean should be corrected by the factor

35 $$\lambda ={(1+(N-\mathrm{1)}\bar{\rho })}^{\mathrm{1/2}}$$

where $\bar{\rho }$ is the average correlation across different observations

36 $$\bar{\rho }=\frac{1}{N(N-\mathrm{1)}}\sum _{i\ne j}{\rho }_{ij}$$

For our application a problem is that we do not know whether the estimators of slopes are correlated. To proceed, we assume that the slopes from different data sets are uncorrelated, and for slopes calculated from different ICA components from the same data set we use the correlation of the envelopes as a proxy. For our data this leads to a correction factor

37 $$\lambda =1.57$$

This might be a crude estimation of the redundancies within the data. However, for the results we show here the standard errors of the mean are tiny compared to the effect size and our main results would be valid even if the correction factor was much bigger.

## Conclusion

We proposed a new method to perform the estimate of long range temporal correlations suggesting modifications of the classical detrended fluctuation analysis. The starting point of the analysis was the observation that in the classical approach the method itself is non-stationary. In general and also for resting state electrophysiological data, which were studied here, this methodological non-stationarity is artificial and, in fact, hampers the analysis.

In particular, one is interested in the slope of detrended (logarithmic) fluctuations as a function of the (logarithmic) scale, i.e. the length of the segment used for detrending. In the classical non-stationary approach such a slope can only be calculated from a linear fit over a wide range of scales. A local calculation of the slope is impossible in that approach due to statistical variations of the results which are much larger than the systematic effects to be studied. We emphasize that this non-stationarity could in principle be avoided within the classical approach using maximally overlapping windows. However, this drastically increases the computational cost and is not done in practice.

To propose a stationary version of DFA, only a minor and almost trivial modification of the detrending procedure was necessary: rather than fitting linear functions in data segments and subtracting those from the data, we only detrend the center for each segment using maximally overlapping segments. The proposed approach is statistically much more stable when the goal is to detect changes of the fluctuations for small changes of the segment length. Second, in the stationary approach linear detrending is equivalent to constant detrending, and the detrending can be simply written as a convolution with a boxcar window, which can then be expressed as a multiplication in the Fourier domain. Moreover, in the representation in the Fourier domain, the length of a window is not necessarily an integer number and we can calculate derivatives of fluctuations with respect to scale, i.e. segment length, analytically. With such expressions we are now in a position to test and specify regions for which that slope is approximately constant and a linear approximation for the fluctuations is justified.

The proposed approach using a boxcar window can be generalized by choosing any reasonable window to detrend data by subtracting a local average. We here suggested a Gaussian window which leads to slightly more stable results than the boxcar window. In either case, formulating the method in the Fourier domain results in very efficient computer code. E.g. the calculation of fluctuations and slopes for 10 minutes of data with a sampling rate of 300 Hz and for 100 different values of the scale *L* takes around 120 milliseconds per channel on an ordinary laptop.

The most prominent application of DFA for EEG and MEG data is the analysis of long-range temporal correlations of amplitudes of oscillations. In particular, the proposed method allows to study effects of a narrow band oscillation on the estimate of the slope of the fluctuations. First of all, we could confirm the general claim that the logarithm of the fluctuations is approximately linear as a function of the logarithm of the scale for relatively large time scales. However, we also found that specifically for the alpha rhythm this is only true for scales above approximately 10 seconds, also when a linear model was found to be the most likely model using BIC or AIC model selection criteria. We therefore want to conclude with the general suggestion to check whether the slopes of interest are constant to reasonable approximation for the range of studied scales.

## Supplementary information

Supplementary Information

## Data Availability

Matlab code and the raw data of all used ICA components containing alpha rhythm are available upon request. The code, named DFA fourier.m, is also included in the “MEG and EEG Toolbox of Hamburg” which can be downloaded at https://www.uke.de/english/departments-institutes/institutes/neurophysiology-and-pathophysiology/research/working-groups/index.html.
