# Supplementary material for: Robust calculation of slopes in detrended fluctuation analysis and its application to envelopes of human alpha rhythms
Source: Sci Rep. 2019 Apr 19;9:6339. doi: 10.1038/s41598-019-42732-7 (PMC6474881; doi:10.1038/s41598-019-42732-7)
Supplement: Supplementary file 1 — Supplementary Information [file 41598_2019_42732_MOESM1_ESM.pdf]

Supplementary Information

Robust calculation of slopes in detrended fluctuation analysis and its application to envelopes of human alpha rhythms

Guido Nolte[1,*], Mohammed Aburidi[2], and Andreas K. Engel[1]

[1]Department of Neurophysiology and Pathophysiology, University Medical Center Hamburg-Eppendorf, Hamburg, Germany
[2]Palestinian Neuroscience Initiative, Al-Quds University, Abu Dis, Jerusalem, Palestine
[*]corresponding author, email: g.nolte@uke.de

We here present the technical details for the estimation of standard errors of the mean for correlated observations. Say, we have N in general correlated observations x_i, with mean subtracted, standard deviations $\sigma =< x_i^2 >^{1/2}$ and correlations

$$\rho_{ij} = \frac{< x_i x_j >}{< x_i^2 >^{1/2} < x_j^2 >^{1/2}} = \frac{< x_i x_j >}{\sigma^2} \tag{1}$$

Specifically, $\rho_{ii} = 1$. Then the variance of the mean of these observations (i.e. the square of the standard error of the mean) reads

$$\begin{aligned} & \langle \left(\frac{1}{N} \sum_{i=1} N x_i \right)^2 \rangle \\ = & \frac{\sigma^2}{N} + \frac{\sigma^2}{N^2} \sum_{i \neq j} \rho_{ij} \\ = & \frac{\sigma^2}{N} (1 + (N-1)\bar{\rho}) \end{aligned} \tag{2}$$

where $\bar{\rho}$ is the average correlation across different observations

$$\bar{\rho} = \frac{1}{N(N-1)} \sum_{i \neq j} \rho_{ij} \tag{3}$$

Thus, taking correlations into account, the standard error of the mean should be corrected with the factor

$$\lambda = (1 + (N-1)\bar{\rho})^{1/2} \tag{4}$$

As a short sanity check we note that for completely redundant observations $\rho = 1$ and $\lambda = \sqrt{N}$ such that the corrected standard error is equal to the standard deviation σ as it should. Also, if we have M independent observations, and each observation occurs twice such that $N = 2M$, then $\bar{\rho} = 1/(N-1)$ and $\lambda = \sqrt{2}$ corresponding to the correct effective number of $N/2$ independent observations.
